# Prediction of absolute risk of acute graft-versus-host disease following hematopoietic cell transplantation

**DOI:** 10.1371/journal.pone.0190610

**Published:** 2018-01-18

**Authors:** Catherine Lee, Sebastien Haneuse, Hai-Lin Wang, Sherri Rose, Stephen R. Spellman, Michael Verneris, Katharine C. Hsu, Katharina Fleischhauer, Stephanie J. Lee, Reza Abdi

**Affiliations:** 1 Kaiser Permanente Division of Research, Oakland, CA, United States of America; 2 Department of Biostatistics, Harvard, T.H. Chan School of Public Health, Boston, MA, United States of America; 3 Center for International Blood and Bone Marrow Transplant Research, Milwaukee, WI, United States of America; 4 Department of Health Care Policy, Harvard Medical School, Boston, MA, United States of America; 5 Center for International Blood and Bone Marrow Transplant Research, Minneapolis, MN, United States of America; 6 Department of Medicine, University of Colorado-Denver, Denver, CO, United States of America; 7 Memorial Sloan Kettering Cancer Center, New York, NY, United States of America; 8 Institute for Experimental Cellular Therapy, University Hospital, Essen, Germany, United States of America; 9 Fred Hutchinson Cancer Research Center, Seattle, WA, United States of America; 10 Transplantation Research Center, Renal Division, Brigham and Women’s Hospital and Children’s Hospital, Boston, MA, United States of America; University of Kentucky, UNITED STATES

## Abstract

Allogeneic hematopoietic cell transplantation (HCT) is the treatment of choice for a variety of hematologic malignancies and disorders. Unfortunately, acute graft-versus-host disease (GVHD) is a frequent complication of HCT. While substantial research has identified clinical, genetic and proteomic risk factors for acute GVHD, few studies have sought to develop risk prediction tools that quantify absolute risk. Such tools would be useful for: optimizing donor selection; guiding GVHD prophylaxis, post-transplant treatment and monitoring strategies; and, recruitment of patients into clinical trials. Using data on 9,651 patients who underwent first allogeneic HLA-identical sibling or unrelated donor HCT between 01/1999-12/2011 for treatment of a hematologic malignancy, we developed and evaluated a suite of risk prediction tools for: (i) acute GVHD within 100 days post-transplant and (ii) a composite endpoint of acute GVHD or death within 100 days post-transplant. We considered two sets of inputs: (i) clinical factors that are typically readily-available, included as main effects; and, (ii) main effects combined with a selection of a priori specified two-way interactions. To build the prediction tools we used the super learner, a recently developed ensemble learning statistical framework that combines results from multiple other algorithms/methods to construct a single, optimal prediction tool. Across the final super learner prediction tools, the area-under-the curve (AUC) ranged from 0.613–0.640. Improving the performance of risk prediction tools will likely require extension beyond clinical factors to include biological variables such as genetic and proteomic biomarkers, although the measurement of these factors may currently not be practical in standard clinical settings.

## Introduction

Allogeneic hematopoietic cell transplantation (HCT) is currently the treatment of choice for a variety of hematologic malignancies and disorders[1, 2]. Unfortunately, acute graft-versus-host disease (GVHD), a debilitating condition associated with significant morbidity, compromised quality of life and mortality remains a frequent complication of HCT[3–8]. To-date, substantial effort has been directed towards identifying factors known before transplant that are associated with increased relative risk of acute GVHD including: patient and donor characteristics, such as the indication for transplant[9], patient age[10] and comorbidities[11], use of an unrelated donor[12], and gender disparity[10]; graft properties, including human leukocyte antigens (HLA) mismatch[13] and immunophenotypic makeup[10]; clinical factors, including transplant conditioning, GVHD prophylaxis strategies[13, 14] and post-transplant infectious events such as cytomegalovirus (CMV) reactivation; genetic factors, including variants of the nucleotide-binding oligomerization domain containing protein 2 (NOD2)[15] and polymorphisms of genes related to interleukin-1 (IL-1)[16]; and plasma protein profiles, including those based on TNF-α[17]. A comprehensive review is given by Harris and colleagues[18].

While clearly important, this body of work has focused on the relative impact of specific risk factors compared to absence of the risk factor. In practice, health care providers, patients and their families are also often interested in understanding and quantifying the absolute risk of acute GVHD for individual patients. Patients facing treatment decisions, for example, would like to know their actual predicted risks of GVHD, not whether they have a “higher” or “lower” risk than others. Furthermore, the quantification of risk could have a number of potentially important uses, particularly towards enabling individualized patient-centered decisions. First, estimating the absolute risk of acute GVHD as a function of the interplay between the characteristics of the patient and potential unrelated donors could help inform decisions about whether to pursue transplantation, which donor to select, and how to perform the transplant. For example, patients at high risk for severe acute GVHD and early mortality may be more circumspect about pursuing transplantation in first remission, or they may be select transplant approaches designed to minimize GVHD, potentially at the cost of greater immunosuppression and higher risk of infections. They may be more interested in clinical trials of novel approaches to prevent GVHD. Conversely, patients whose risk of severe acute GVHD is low may not require aggressive immunosuppression. From a research perspective, the quantification of absolute risk could be used as an inclusion criterion for clinical trials to select appropriate participants based on risk profile.

For the most part studies seeking to develop and validate prediction tools for absolute risk have focused on outcomes, particularly mortality, following the onset of acute GVHD[16, 17, 19]. Substantially less attention has been paid to the quantification of absolute risk of acute GVHD for a patient who is about to undergo or who has just undergone HCT. Notable exceptions include recent efforts to develop prediction tools based on proteomic biomarker panels[20, 21]. These studies, however, rely on measurements that may be difficult to obtain in typical clinical settings and/or are measured after the transplant has already occurred[22–25], making them unsuitable for pre-transplant risk prediction and selection of GVHD prophylaxis. In this work, we seek to develop and evaluate a risk prediction tool for acute GVHD that could be readily-implemented, and therefore broadly useful, by focusing on patient-, donor-, transplant- and graft-specific factors that are typically available in standard clinical settings. Towards developing risk prediction tools, researchers have at their disposal a vast number of options[26]. The statistical framework we employ is the recently developed super learner ensemble learning framework[27]. As we elaborate upon, the super learner works by combining predictions obtained from a range of algorithms/methods, each of which may be used to construct a prediction tool, to form a single overarching prediction tool. Through theoretical work and simulations, the super learner framework has been shown to enjoy a number of optimality properties, including that the final prediction tool outperforms or does no worse than any of the component algorithm/methods, and has been successfully used in a broad range of clinical settings[28–31].

## Methods

### Patients

This is a multi-institutional study based on data from the Center for International Blood and Bone Marrow Transplant Research (CIBMTR), a collaboration between the National Marrow Donor Program and the Medical College of Wisconsin representing a worldwide network of transplant centers that contribute detailed data on HCT. Studies conducted by the CIBMTR are performed in compliance with all applicable federal regulations pertaining to the protection of human research participants. Protected Health Information used in research is collected and maintained in CIBMTR’s capacity as a Public Health Authority under the HIPAA Privacy Rule.

Data were extracted from the CIBMTR databases for 10,178 patients who underwent first allogeneic HLA-identical sibling or unrelated donor HCT between January 1999 and December 2011 for treatment of acute myeloid leukemia (AML), acute lymphoblastic leukemia (ALL), myelodysplastic syndrome (MDS) or chronic myeloid leukemia (CML), using either bone marrow or peripheral blood stem cells combined with myeloablative or reduced intensity/non-myeloablative conditioning. For each patient, HLA identical sibling match assessments were performed per center practice. For patients with an unrelated donor, HLA matching was determined at high resolution for HLA-A, B, C, DRB1 and DQB1 through retrospective typing of stored pre-transplant samples and/or reported by the transplant center and match assessment performed per CIBMTR criteria[32]. Infection prophylaxis and treatment were managed according to each institution’s standard practice guidelines. Prior to analyses we excluded patients with missing values for any of the following: disease status, donor-recipient sex matching, conditioning intensity and GVHD prophylaxis. This resulted in a final analytic sample of 9,651 patients. Access to the dataset may be obtained from the CIBMTR after execution of a data use agreement.

### Outcomes

The primary outcome of interest was the binary endpoint indicating whether the patient had a diagnosis of grade III or IV acute GVHD within 100 days of transplantation[33]. In secondary analyses, since early death could prevent the development of acute GVHD, we also considered a composite binary endpoint indicating whether the patient was diagnosed with acute GVHD grades III-IV or died within 100 days of HCT.

This analysis used patients reported on Case Report Forms (CRFs) and excluded patients reported solely on Transplant Essential Data (TED) abbreviated forms. Only CRFs captured detailed information about the timing of acute GVHD and severity of individual organ systems, allowing application of a standardized algorithm that calculates the overall acute GVHD grade. CIBMTR selects patients to be reported on CRF or TED forms according to a central algorithm based on patient and transplant characteristics, not patient outcomes.

### Risk factors

In developing the risk prediction tools we focused on factors that are typically available to health care providers who oversee the care of patients undergoing HCT and that have been identified in other studies of GVHD. These included: patient gender, patient age, disease type (AML, ALL, MDS or CML), disease status (early, intermediate or advanced), donor-patient female-male sex-mismatch, patient-donor CMV serology match, patient-unrelated donor HLA-compatibility (8/8 or 7/8 HLA-matched), graft type (bone marrow or peripheral blood), conditioning intensity (myeloablative or reduced intensity/non-myeloablative), GVHD prophylaxis regimen, in-vivo T-cell depletion (no or yes), and Karnofsky score. All variables were available in categorized form, including nominally continuous variables such as patient age (<10, 10–19, 20–29, 30–39, 40–49, 50–59, ≥60) and Karnofsky score (<90%, ≥90%).

For both the primary and secondary outcomes we developed two sets of prediction tools. The first solely considered main effects for each of the risk factors. The second set additionally considered a series of two-way interactions that were identified *a priori* as being of potential predictive value based on clinical considerations. These included interactions between: HLA-compatibility and patient/disease characteristics (gender, age, disease type and disease status); HLA-compatibility and donor-patient matching variables (sex, CMV); HLA-compatibility and transplant variables (graft type, conditioning intensity, prophylaxis regimen, use of in vivo T-cell depletion); patient age and donor-patient matching variables (sex, CMV); patient age and the use of in vivo T-cell depletion; disease type and donor-patient matching variables (sex, CMV); disease type and transplant variables (graft type, conditioning intensity, prophylaxis regimen, use of in vivo T-cell depletion); disease status and donor-patient matching variables (sex, CMV); and disease status and transplant variables (graft type, conditioning intensity, prophylaxis regimen, use of in vivo T-cell depletion). Information on HLA-DP typing was not available for the full cohort, thus was not included as a potential predictor.

In general, missing data among the factors we consider for inclusion as predictive factors was minimal; 5.7% of patients had a missing value for Karnofsky performance status, while 2.4% of patients had missing data on the patient-donor CMV serology match. For both of these variables, our strategy for addressing missing values was to code an additional “missing” category.

### Statistical analysis

Since all risk factor variables were available in categorical form, the sample population was initially described using frequency counts and corresponding percentages. Additionally, prior to conducting our main analyses, we conducted a series of analyses examining univariate (i.e. unadjusted) associations between each of the risk factors and the two binary outcomes.

### Development of the prediction tools

To develop the prediction tools we employed the super learner, a recently developed ensemble learning framework[27, 28]. Briefly, use of the super learner framework consists of two stages. At the first stage a series of prediction tools are developed using a set of candidate algorithms/methods. In our implementation we considered the following algorithms/methods: standard logistic regression[34], logistic regression via the lasso[35], generalized boosted regression[36], generalized additive regression[37], polynomial spline regression[38], Bayesian additive regression trees[39], ridge regression[40], elastic net regularization[41], and neural networks[42]. For each of these algorithm/methods, patient-specific predictions were obtained via 10-fold cross-validation[26]. In principle, analysts using the super learner framework may consider any number of algorithms/methods that could individually be used to develop a risk prediction tool for inclusion in the set of candidates. Our choice for the candidate set was guided by our prior experience in implementing the super learner, through consideration of the pros and cons of each algorithm/method as reported in the literature, and through consideration of the computational burden associated with adding more algorithms/methods.

At the second stage a logistic regression of the binary outcome (i.e. acute GVHD or the composite outcome of acute GVHD or death) is fit with the patient-specific cross-validated predictions from the individual candidate algorithms/methods used as inputs. The estimated coefficients from this logistic regression are then used to construct a final weighted combination that constitutes the super learner function; the coefficient weights serve to either increase or decrease the influence of any individual candidate algorithm/method. From a theoretical perspective, the super learner has been shown to be optimal in the sense that predictions from the final tool are guaranteed to perform at least as well asymptotically (i.e. as the sample size grows) as the predictions from the best individual candidate algorithm/method[27]. Furthermore, in constructing a weighted score using predictions from the individual algorithms/methods, the super learner has the advantage of not relying on any single individual algorithm/method that may perform well in some settings but not in others.

### Evaluation of predictive performance

To evaluate predictive performance of the predictive tools we calculated the receiver operating characteristic (ROC) curve as well as three numerical criteria that are relevant when considering whether the model can be used to guide patient management: calibration, discrimination and risk stratification[43, 44]. Calibration assesses the goodness-of-fit of the predicted values by initially stratifying the patients on the basis of their predicted risk using pre-specified risk intervals. Within each interval, the proportion of patients who actually experienced the outcome is then compared to the mid-point of the risk interval. If these two numbers align across all intervals, the tool is regarded as being well calibrated. The second criterion, discrimination, summarizes the prediction tool’s ability to correctly classify events and non-events. Typically, discrimination is summarized via the area under the curve (AUC) statistic. Towards calculation of AUC, one would ideally evaluate predictive performance on an independent sample. This could be accomplished by randomly splitting the available data in two (i.e. one part for model building and another for evaluation), although this strategy is known to be inefficient[45]. To avoid loss of information, we used the entire sample of 9,651 patients to develop the final prediction tools and then based the calculation of AUC based on 10-fold cross-validation[26]. For comparison, we also computed the “apparent” AUC in which the predictive performance was evaluated using the original sample. The final criterion, risk stratification, provides a means to evaluate the contribution of the interaction terms. Briefly, for a patient’s predicted risk to be useful it should ideally indicate a clear action or decision. This most naturally occurs when patients have a predicted risk that is either small or large (i.e. close to 0.0 or close to 1.0). Risk stratification summarizes this notion in our setting by comparing the number of patients allocated to the extremes of the risk distribution based on the main effects and interaction terms prediction tool to corresponding number based on the main effects only prediction tool. Finally, we estimated the Kaplan-Meier estimate of the survivor curve associated with time to acute GVHD based on the main effects only super learner prediction tool, stratifying patients by their predicted risk into three groups: low risk, 0–10%; medium risk, 11–25%; high risk >25%.

### Illustration of clinical utility

Finally, we illustrate how the risk prediction tools could be used in clinical practice. Specifically, we consider two clinical scenarios for a hypothetical 50-year-old male patient with a Karnofsky score of 90% and positive CMV serology, who was diagnosed with intermediate risk AML and is in second complete remission. In the first scenario this patient is about to undergo a transplant from his CMV+ HLA-identical brother using myeloablative conditioning. In the second scenario, he will instead receive reduced intensity conditioning because of co-morbidities of diabetes, prior colon cancer, and moderate pulmonary dysfunction. In this scenario, an 8/8 unrelated donor with CMV negative serology has been identified. We illustrate the range of estimated GVHD rates considering graft type, T cell depletion and GVHD prophylaxis, all factors controlled by the transplant center.

Throughout, all statistical analyses were conducted in the R statistical environment[46] (version 3.2.2). The code used to conduct the analyses is provided in online Supplementary Materials.

## Results

The first column of Table 1 presents demographic, clinical and donor for all 9,651 patients in the study sample. The majority of patients were male (55.6%), with most being between 20–59 years of age at the time of HCT (75.2%). Furthermore, approximately half of the patients underwent HCT for AML (51.0%) and transplantation was performed in an early or intermediate disease state (74.5%). The vast majority of patients (83.3%) received their graft from either an HLA-identical sibling or an 8/8 HLA compatible unrelated donor, with approximately two-thirds of patients receiving a peripheral blood graft (64.7%). Finally, just over three-quarters of patients underwent myeloablative conditioning (80.1%).

**Table 1:** Patient and donor characteristics for 9,561 patients who underwent HCT between 01/1999-12/2011 for treatment of AML, ALL, MDS or CML. Also shown are unadjusted event rates and results from univariate logistic regressions (OR = odds ratio; CI = confidence interval) for the two binary outcomes of acute GVHD grades III-IV within 100 days and a composite endpoint of the first of death or acute GVHD grades III-IV within 100 days.

			Acute GVHD grades III-IV				Composite endpoint			
			Event rate	Univariate Logistic Regression			Event rate	Univariate Logistic Regression		
	N	%	(%)	OR	95% CI	p-value	(%)	OR	95% CI	p-value
**Total**	9,651									
**Gender**										
Male	5,366	55.6	18.6	1.00		0.006	28.1	1.00		0.632
Female	4,285	44.4	16.4	0.86	(0.77, 0.96)		27.7	0.98	(0.89, 1.07)	
**Age, years**										
Younger than 10	653	6.8	12.7	0.66	(0.5, 0.84)	0.005	18.8	0.57	(0.45, 0.7)	<0.001
10–19	1,162	12.0	16.9	0.91	(0.75, 1.1)		25.2	0.83	(0.71, 0.98)	
20–29	1,572	16.3	19.4	1.08	(0.92, 1.28)		29.0	1.01	(0.87, 1.16)	
30–39	1,581	16.4	17.8	0.98	(0.82, 1.16)		27.5	0.93	(0.81, 1.08)	
40–49	2,095	21.7	18.2	1.00			29.0	1.00		
50–59	2,008	20.8	18.3	1.01	(0.86, 1.18)		30.8	1.11	(0.97, 1.26)	
60 or older	580	6.0	15.0	0.79	(0.61, 1.02)		28.1	0.97	(0.79, 1.19)	
**Disease type**										
AML	4,919	51.0	16.2	1.00		<0.001	27.0	1.00		0.127
ALL	2,071	21.5	17.0	1.06	(0.93, 1.22)		28.4	1.07	(0.96, 1.2)	
CML	1,525	15.8	21.1	1.39	(1.2, 1.6)		28.5	1.08	(0.95, 1.22)	
MDS	1,136	11.8	20.2	1.31	(1.11, 1.55)		30.2	1.17	(1.02, 1.35)	
**Disease status**										
Early	4,873	50.5	16.4	1.00		0.002	23.0	1.00		<0.001
Intermediate	2,316	24.0	18.1	1.13	(0.99, 1.29)		27.8	1.30	(1.16, 1.46)	
Advanced	2,462	25.5	19.6	1.25	(1.1, 1.41)		37.8	2.05	(1.85, 2.28)	
**Karnofsky score**										
Less than 90%	2,723	28.2	18.7	1.10	(0.98, 1.24)	0.243	33.9	1.52	(1.38, 1.68)	<0.001
90–100%	6,382	66.1	17.3	1.00			25.3	1.00		
Missing	546	5.7	16.8	0.97	(0.77, 1.22)		28.9	1.22	(1, 1.47)	
**D-R sex match: F-M**										
No	7,732	80.1	17.3	1.00		0.097	27.8	1.00		0.520
Yes	1,919	19.9	18.9	1.11	(0.98, 1.27)		28.5	1.04	(0.93, 1.16)	
**D-R CMV match**										
-/-	2,594	26.9	18.9	1.16	(1.01, 1.33)	0.239	28.1	1.06	(0.94, 1.19)	0.584
-/+	2,741	28.4	17.1	1.02	(0.89, 1.18)		28.8	1.10	(0.98, 1.23)	
+/-	1,135	11.8	18.1	1.10	(0.92, 1.32)		28.2	1.06	(0.91, 1.24)	
+/+	2,953	30.6	16.7	1.00			27.0	1.00		
Missing	228	2.4	18.9	1.16	(0.81, 1.62)		27.2	0.98	(0.71, 1.32)	
**HLA compatibility**										
HLA-Identical Sibling	3,941	40.8	13.5	1.00		<0.001	21.8	1.00		<0.001
8/8	4,100	42.5	19.1	1.51	(1.34, 1.7)		30.0	1.58	(1.43, 1.75)	
7/8	1,610	16.7	23.8	1.99	(1.72, 2.31)		37.8	2.24	(1.97, 2.54)	
**Graft type**										
Bone marrow	3,405	35.3	16.2	0.85	(0.76, 0.95)	0.005	27.5	0.96	(0.87, 1.05)	0.387
Peripheral blood	6,246	64.7	18.4	1.00			28.2	1.00		
**Conditioning intensity**										
Myeloablative	7,732	80.1	18.2	1.00		0.001	28.1	1.00		0.436
Reduced intensity/non-myeloablative	1,919	19.9	15.1	0.80	(0.69, 0.91)		27.1	0.96	(0.85, 1.07)	
**GVHD prophylaxis**										
Ex-vivo TCD/CD34 Selection	523	5.4	12.2	0.69	(0.52, 0.9)	<0.001	26.6	1.03	(0.83, 1.26)	<0.001
Post-HCT Cy	49	0.5	22.4	1.42	(0.69, 2.71)		40.8	1.82	(1, 3.22)	
Tac+MTX+/-others	3,686	38.2	16.9	1.00			26.0	1.00		
Tac+/-others	1,686	17.5	20.3	1.26	(1.08, 1.45)		32.1	1.35	(1.19, 1.54)	
CSA+MTX+/-others	2,809	29.1	17.0	1.01	(0.88, 1.15)		26.8	1.03	(0.92, 1.15)	
CSA+/-others	898	9.3	20.3	1.25	(1.04, 1.5)		32.0	1.34	(1.14, 1.57)	
**In vivo T-cell depletion**										
No	7,538	78.1	18.6	1.00		<0.001	28.2	1.00		0.393
Yes	2,113	21.9	14.2	0.73	(0.64, 0.83)		27.1	0.95	(0.86, 1.06)	

Of the 9,651 patients in the study, 1,701 (17.6%) developed acute GVHD grades III-IV, while 1,477 (15.3%) died within 100 days. Furthermore, 2,679 (27.8%) experienced at least one of these events before 100 days, while 499 (5.2%) experienced both. Most of the factors we considered for inclusion in the risk prediction tools were significantly associated with risk of acute GVHD within 100 days in univariate analyses (Table 1), although determining the clinical implications of specific estimated associations should proceed with caution. In contrast, notwithstanding the increased event rate, only age, disease status, Karnofsky score, HLA compatibility, GVHD prophylaxis regimen and conditioning intensity were significantly associated in unadjusted analyses with the composite endpoint of severe acute GVHD and/or 100 day mortality in univariate analyses.

**Fig 1:**
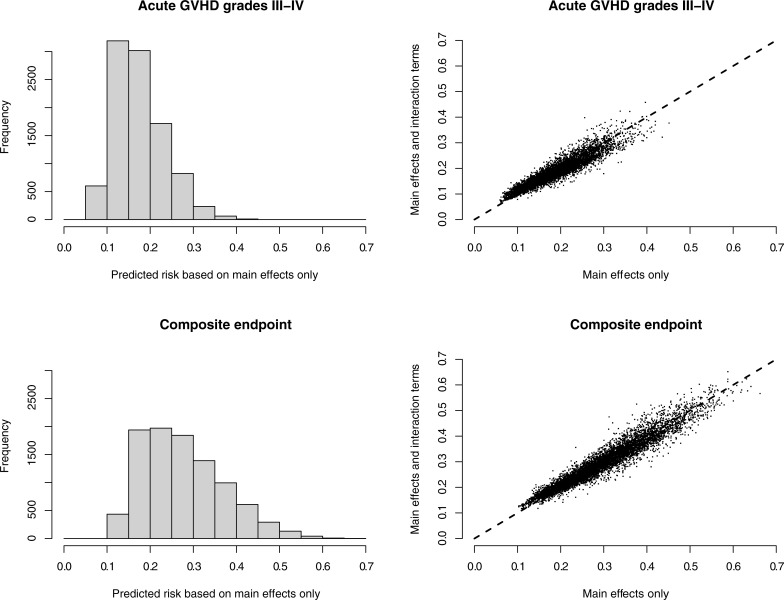
Risk predictions from super leaner analyses for 9,651 patients at risk for: (i) acute GVHD within 100 days, and (ii) the composite endpoint of acute GVHD and death within 100 days. For each outcome risk predictions are presented for two tools: one based solely on main effects for risk factors considered and another based on main effects and select two-way interactions.

Fig 1 provides a summary of the risk predictions obtained from the four super learner tools. From top-left panel of Fig 1, the estimated probability of acute GVHD within 100 days based solely on main effects ranged between 0.06 and 0.39, with a median of 0.17 and an inter-quartile range (IQR) of (0.14, 0.20). Permitting the inclusion of interaction terms did not meaningfully change the predictions, as evidenced by the strong correlation between the two sets (top-right panel of Fig 1). From the bottom-left panel the median predicted risk for the composite endpoint based on the main effects only tool was 0.27 with a range of 0.03 to 0.65 and IQR of (0.21, 0.34). As with acute GVHD within 100 days, the inclusion of interaction terms did not meaningfully change the risk predictions for the composite endpoint (bottom-right panel of Fig 1).

Table 2 shows that each of the four super learner risk scores are well-calibrated; within each stratum defined by predicted risk the percentage of patients who actually experienced the endpoint is consistent with the strata limits. For example, among the 6,714 patients whose predicted risk for acute GVHD based on the main effects only tool was between 10% and 20%, the percentage of patients who actually experienced an acute GVHD event was 14.4%.

**Table 2:** Summary of calibration and risk stratification performance for four super learner risk prediction tools.

	**Risk strata based on predicted probabilities**					
**Acute GVHD grades III-IV**	(0, 10]	(10, 20]	(20, 30]	>30		
*Main effects only*						
Number in strata	317	6714	2529	91	-	-
Percent in strata	3.3	69.6	26.2	0.9	-	-
Percent with diagnosis	7.3	14.4	26.5	46.2	-	-
*Main effects and interactions*						
Number in strata	521	6258	2568	304	-	-
Percent in strata	5.4	64.8	26.6	3.1	-	-
Percent with diagnosis	6.3	14.5	25.2	37.8	-	-
	**Risk strata based on predicted probabilities**					
**Composite end point**		≤ 20	(20, 30]	(30, 40]	(40, 50]	>50
*Main effects only*						
Number in strata	-	2205	3735	2591	934	186
Percent in strata	-	22.8	38.7	26.8	9.7	1.9
Percent with diagnosis	-	13.3	24.4	35.6	47.1	61.3
*Main effects and interactions*						
Number in strata	-	2315	3626	2524	1005	181
Percent in strata	-	24	37.6	26.2	10.4	1.9
Percent with diagnosis	-	13.1	25	35.1	47.8	57.5

**Fig 2:**
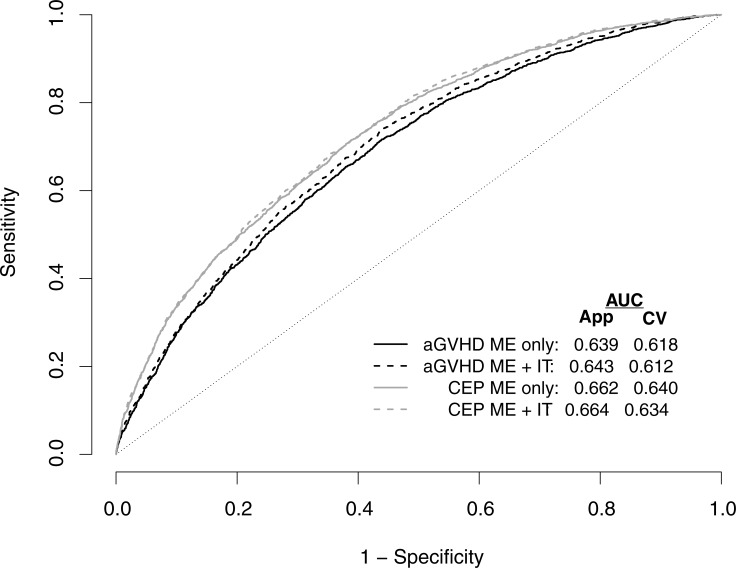
Receiver operating characteristics curves corresponding to super learner predictive tools for 9,651 patients at risk for: (i) acute GVHD within 100 days, and (ii) the composite endpoint (CEP) of acute GVHD and death within 100 days. For both outcomes, two prediction tools were developed: one based solely on main effects (ME only) for risk factors considered and another based on main effects and select two-way interactions (ME + IT). Also shown are apparent (App) and cross-validated (CV) area-under-the-curve (AUC) statistics.

**Fig 3:**
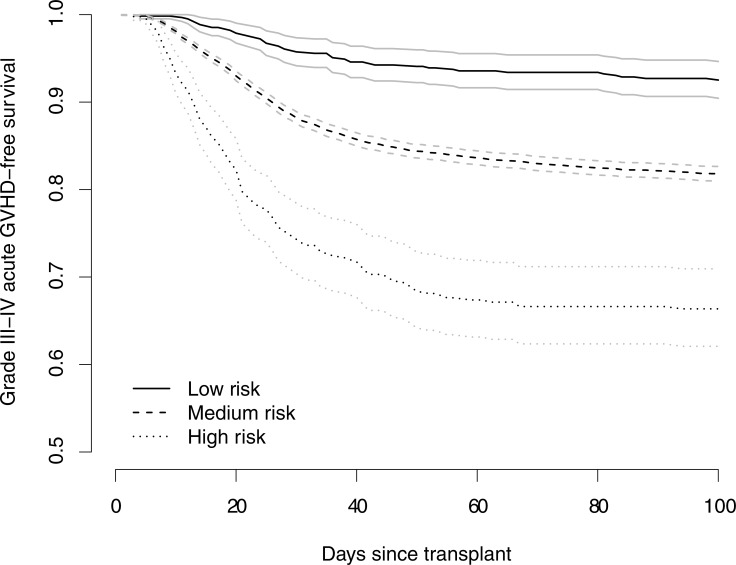
Kaplan-Meier estimates and pointwise 95% confidence intervals for grade III-IV acute GVHD-free survival within 100 days among 9,651 patients who underwent who underwent first allogeneic HLA-identical sibling or unrelated donor HCT for treatment of a hematologic malignancy, stratified by risk group according to the super learner prediction tool based solely on main effects: low risk, 0–10%; medium risk, 11–25%; high risk >25%.

Figs 2 and 3 and Table 3 summarize the discriminatory performance of the four super learner prediction tools. The cross-validated AUC for the super learner prediction tool for acute GVHD based solely on main effects is 0.618; the corresponding cross-validated AUC based on main effects and interactions terms is 0.612 (Fig 2). Furthermore, the cross-validated AUC for the super learner prediction tool for the composite endpoint based solely on main effects is 0.640; the corresponding cross-validated AUC based on main effects and interactions terms is 0.634. When stratified on the basis of predicted risk from the super learner tool for acute GVHD based solely on main effects, patients exhibited increasingly poor outcomes across the low, medium and high risk groups (Fig 3). Finally, as anticipated by theoretical considerations, the super learner outperformed or did no worse than each of the component algorithm/methods (Table 3).

**Table 3:** Apparent and cross-validated area-under-the-curve (AUC) statistics for four super learner risk prediction tools, as well as for each of the component algorithms/methods considered in the implementation of the super learner.

	Acute GVHD grades III-IV				Composite end point			
	Main effects only		Main effects and interactions		Main effects only		Main effects and interactions	
	Apparent	Cross-	Apparent	Cross-	Apparent	Cross-	Apparent	Cross-
		validated		validated		validated		validated
Logistic regression	0.630	0.617	0.660	0.595	0.641	0.632	0.667	0.620
Logistic regression + Lasso	0.629	0.617	0.638	0.613	0.641	0.633	0.649	0.628
Generalized boosted regression	0.644	0.618	0.654	0.609	0.653	0.637	0.657	0.630
Generalized additive models	0.630	0.617	0.660	0.595	0.641	0.632	0.667	0.620
Polynomial spline regression	0.500	0.545	0.618	0.500	0.640	0.624	0.628	0.623
Bayesian additive regression trees	0.645	0.619	0.649	0.615	0.660	0.641	0.658	0.636
Ridge regression	0.630	0.617	0.648	0.600	0.641	0.632	0.658	0.625
Elastic net	0.629	0.617	0.639	0.611	0.641	0.633	0.649	0.628
Neural network (hidden layers = 2)	0.500	0.500	0.500	0.500	0.667	0.570	0.716	0.561
Neural network (hidden layers = 5)	0.500	0.503	0.500	0.500	0.699	0.578	0.775	0.534
Super learner	0.639	0.618	0.643	0.612	0.662	0.640	0.664	0.634

Consistent with the observations from Fig 1, inclusion of interaction terms in the prediction tools did not meaningfully improve risk stratification (Table 2). For the acute GVHD outcome 4.2% of patients were allocated to the lowest and highest risk strata based on the main effects only super learner; based on the main effects and interaction terms super learner only 8.4% were allocated to these strata. Similarly, while 24.7% of patients were allocated to the lowest and highest risk strata for the composite endpoint based on the main effects only super learner, only 25.9% were allocated to these strata based on the main effects and interaction terms super learner.

Finally, we calculated the predicted risk for acute GVHD within 100 days of HCT for the hypothetical 50-year-old man based on the main effects only prediction tool. In particular, if the patient underwent transplant from his CMV-positive, HLA-identical brother using peripheral blood and Tac+MTX and no in vivo T-cell depletion, his predicted risk of grade III-IV acute GVHD would be 14.6%. If he underwent the same transplant but his brother donated bone marrow instead, his risk would be 12.2% or if peripheral blood was used but in vivo T cell depletion was added, his risk would be 11.7%. If he received reduced intensity conditioning and peripheral blood from an 8/8 CMV-negative female donor with Tac+MTX GVHD prophylaxis and no in vivo T-cell depletion, his risk would be 16.6%. If GVHD prophylaxis was switched to tacrolimus and mycophenolate mofetil without methotrexate, his risk would be 19.4%. Other patients getting similar transplants as this last patient might be encouraged to participate in a novel GVHD prevention trial and the trial would need far fewer patients because of the higher baseline risk. In contrast, those getting bone marrow from HLA-identical siblings would have less to gain from more aggressive immunosuppression and showing a benefit with the intervention would require a prohibitive sample size.

## Discussion

As the number of patients undergoing HCT increases, the burden of severe acute GVHD will also increase. The past decade has witnessed significant shifts towards matching unrelated donors and patients on the basis of HLA, the prime determinant of compatibility. This standardization of pre-transplant donor-recipient matching in combination with better supportive care has significantly improved outcomes[47, 48]. Despite HLA matching, however, GVHD remains a serious and frequent complication of HCT with approximately 50% of patients developing some acute GVHD, of which a third is considered severe. As such, while overall survival is arguably the most important clinical outcome, there is a significant need for validated prediction tools that informs a patient of their absolute risk of acute GVHD, and that be used as a basis for making treatment and monitoring strategy decisions. In this paper we address this gap. Crucially, towards ensuring that the prediction tools could be easily implemented, we chose to focus on factors that are readily-available in clinical settings.

The key strengths of this paper are two-fold. First is that the available data consisted of detailed clinical information on a large sample that reflects real-world heterogeneity in patients who undergo HCT. Specifically, the data are representative of the broad range of patient-donor characteristics observed in clinical settings as well as the diverse ways in which patients are treated prophylactically and post-transplant. In this sense, the final predictive models can be viewed as being relevant to real-world clinical settings. Furthermore, that the sample was large also permitted the inclusion of interaction terms between predictive factors which, in turn, introduced flexibility in how a given factor might influence a patients risk.

A second strength of the paper is our use of modern methods for the development of risk prediction models, currently a major area of research in the statistical and machine learning literature. Our choice to use the super learner framework was driven by both theoretical considerations and simulations which show that it outperforms standard techniques in many common data settings, including when there are a small to moderate number of moderate-sized effects and a large number of small effect sizes[27]. These features are likely present in heterogeneous clinical populations, such as the HCT population we consider, and when the goal is to predict a clinically complex outcome, such as acute GVHD. Furthermore, a central appeal of the super learner is that it does not require analysts to choose and rely on a single algorithm/method; the final prediction tool can therefore be viewed as being robust to the model misspecification. One potential drawback of this robustness, however, is that the framework does not provide a simple characterization of the influence or statistical significance of any single input or predictive factor. This is in contrast to, say, multivariate logistic regression wherein the effect of a single factor is quantified via an odds ratio. While such simple characterizations can be useful, especially if interest lies with the relative impact of a specific factor, the philosophy of the super learner is not to identify whether and how individual factors are predictive but rather to provide a flexible framework within which the impact of any factor is not constrained. In a multivariate logistic regression model, for example, a risk factor may only influence the prediction through the strength of the odds ratio association. In contrast, depending on the chosen set of candidate algorithms/methods, any given factor may influence the final super learner through one or many mechanisms.

From a clinical perspective, the predictive performance of the four super learner models is comparable to that reported by Sorror and colleagues who investigated the value of a pre-transplant HCT comorbidity index, HCT-CI, in predicting the development of acute GVHD following HCT[11]; in particular, they report an AUC of 0.64 associated with prediction based on HCT-CI. In principle, it is possible that including HCT-CI in the pool of factors we considered may have yielded predictive tools with superior performance. Data for this instrument, however, has only recently been collected by CIBMTR and could therefore not be included. Moreover, the comparability of the AUCs from our study and the Sorror study suggests that any improvements would be minimal.

Moving forward, our results suggest that additional efforts at exploring alternative statistical methods and/or flexible approaches to modeling, including interaction terms, are unlikely to be worthwhile. In particular, while such efforts may lead to closer representations of the underlying data generating mechanism (which prediction models are, in some sense, trying to mimic), there is a limit to how much information one can extract from any given set of variables. Instead, as others have argued[19–21, 49], we believe that the strategy with the greatest potential to improve performance is one that focuses on building prediction tools that jointly consider clinical factors with recently-identified genetic factors and proteomic biomarkers[18]. While this represents a natural next step, it is important to note that the implementation of such prediction tools in standard clinical settings may be limited if these measures are not readily-available or routinely collected. This may change, however, as high-throughput proteogenomic technologies advance and become affordable.
